# Risk factors for Luminal A ductal carcinoma *in situ* (DCIS) and invasive breast cancer in the Carolina Breast Cancer Study

**DOI:** 10.1371/journal.pone.0211488

**Published:** 2019-01-25

**Authors:** Lindsay A. Williams, Patricia Casbas-Hernandez, Hazel B. Nichols, Chiu Kit Tse, Emma H. Allott, Lisa A. Carey, Andrew F. Olshan, Melissa A. Troester

**Affiliations:** 1 Department of Epidemiology, Gillings School of Global Public Health, University of North Carolina at Chapel Hill, Chapel Hill, United States of America; 2 Department of Nutrition, Gillings School of Global Public Health, University of North Carolina at Chapel Hill, Chapel Hill, United States of America; 3 Lineberger Comprehensive Cancer Center, University of North Carolina at Chapel Hill, Chapel Hill, United States of America; 4 Department of Pathology and Laboratory Medicine, School of Medicine, University of North Carolina at Chapel Hill, Chapel Hill, United States of America; University of Kentucky, UNITED STATES

## Abstract

**Purpose:**

Invasive breast cancers are thought to arise from *in situ* lesions, but some ductal carcinoma *in situ* (DCIS) are indolent with low likelihood of progressing to invasive carcinoma. Comparison of risk factor associations between DCIS and invasive disease may elucidate which factors influence early versus late stages of carcinogenesis. Therefore, we determined whether there were differences in risk factor profiles for screen-detected DCIS and invasive breast cancer among Luminal A lesions.

**Methods:**

We conducted a case-control analysis using data from the Carolina Breast Cancer Study (1993–2001). Analyses were restricted to Luminal A tumors and screen-detected tumors among mammography-eligible women, to limit confounding by mode of detection (N = 108 DCIS; N = 203 invasive). Logistic regression was used to estimate odds ratios (OR) and 95% confidence intervals (CI) for associations between risk factors and lesion type.

**Results:**

In stratified analyses, we observed qualitative differences in the direction of association for ever smoking, obese BMI, high waist-to-hip-ratio (WHR), and ≥10 years of oral contraceptive use between DCIS and invasive disease. Breastfeeding was inversely associated with invasive disease and was not associated with DCIS. Interaction tests for risk factor associations between Luminal A DCIS and invasive breast cancer were not statistically significant (p>0.05).

**Conclusions:**

Among Luminal A tumors, established breast cancer risk factors may exert stronger effects on progression of early lesions to invasive disease, with lesser effects on risk of DCIS.

## Introduction

Breast carcinogenesis theories state that invasive breast cancers may arise from pre-invasive lesions, such as ductal carcinoma *in situ* (DCIS), which has increased in incidence in the last two decades due to increased screening.[1, 2] DCIS lesions harbor molecular changes that characterize invasive breast cancers; yet not all DCIS progress into invasive disease.[3–5] Anderson *et al*. (2004) performed a detailed analysis of incident DCIS and invasive breast cancer to assess the hypothesis that all invasive cancers have an *in situ* phase. They concluded that incidence patterns observed in SEER (1973–2000) contradict the idea that carcinoma *in situ* (CIS) is an obligate step in progression.[2] Others have argued that CIS is not an obligate precursor of invasive breast cancer due to a low probability of developing invasive breast cancer after CIS diagnosis[6], variable rates of diagnoses of invasive breast cancer after DCIS ranging from 14–53%[7–10], and autopsy studies reporting that 6–18% of the population may have undetected DCIS at the time of death.[11, 12] Together these findings suggest that DCIS and invasive breast cancer may represent distinct disease pathways, leading some to argue for a narrower definition of breast cancer (to exclude DCIS).[13] It is clear that more research is needed to distinguish indolent DCIS from lesions that will progress into invasive breast cancers.

The aim of this study was to compare risk factor profiles for screen-detected DCIS and invasive breast cancer among Luminal A lesions to try and elucidate the pathways that may be important in etiology and progression of DCIS. Using the Carolina Breast Cancer Study, we restricted analyses to Luminal A cases, which comprise a majority of DCIS cases[14], because breast cancer represents multiple diseases (i.e. Luminal A, Luminal B, Basal-like, HER2-enriched) with distinct risk factors[15–17] and the frequency of each subtype varies between DCIS and invasive breast cancers due to length bias.[18–21] Second, the vast majority of DCIS are detected by mammography, while invasive breast cancers are more often symptomatic. Therefore, we further restricted analyses to screen-detected, Luminal A lesions in an effort to isolate the influence of mode of detection and tumor heterogeneity. By controlling these variables, analyses comparing risk factors for DCIS and invasive disease can shed light on the role of various exposures in breast cancer progression.

## Methods

### Study population

The Carolina Breast Cancer Study (CBCS) is a population-based study of African American and Non-African American (98% Caucasian, referred to as White) women from 24 counties of central and eastern North Carolina.[15, 22] The current analysis includes women from Phase 1 (1993–1996) and Phase 2 (1996–2001). Cases of invasive breast cancer were enrolled in Phases 1 and 2. Women with carcinoma *in situ* (CIS) (both Lobular and Ductal Carcinoma *In Situ*) were enrolled in Phase 2. African American and younger women (age <50) with invasive breast cancer were oversampled using randomized recruitment[19], but there was no oversampling by race or age for CIS cases. All participants provided informed consent as approved by the Institutional Review Board at the University of North Carolina, School of Medicine, which also provided approval for this study.

Cases were identified using rapid case ascertainment in collaboration with the North Carolina Central Cancer Registry. Women between the ages of 20–74, diagnosed with primary invasive breast cancer or DCIS, and who completed full questionnaires (described elsewhere [15, 22]) were eligible. Controls were identified from North Carolina Division of Motor Vehicles lists (age <65) and the Health Care Financing Administration (age ≥65) and were frequency matched to cases by 5-year age and race. Separate control groups were selected for the invasive breast cancer and CIS cases, taking into account the different sampling schema for the two case groups. We restricted the main analysis to women eligible for routine screening mammography (aged ≥40 years, as actual screening adherence for each control was unknown), who had a screen-detected lesion (N = 203), and Luminal A tumors (defined as HER2- and ER+ and/or PR+, N = 547). Invasive controls (N = 1,367) aged ≥40 years who completed full questionnaires were included. A total of 503 CIS cases were enrolled in CBCS. CIS inclusion criteria were similar to those for invasive breast cancer and resulted in the inclusion of 108 cases in our main analyses: questionnaire completion, histological marker information (explained elsehwere[15, 19]), aged ≥40 years, screen-detected lesions and Luminal A (defined as HER2- and ER+, N = 139, representing 62% of all DCIS cases with information on histological markers). A total of 424 CIS controls (aged≥40 years) completed the full questionnaire.

### Immunohistochemistry

Cases granted permission to obtain medical records, pathology reports, and tumor blocks.[15] Briefly, for invasive breast cancer, estrogen receptor (ER) and progesterone receptor (PR) status was obtained from the medical records in 80% of cases (positive, negative; percent positivity was not available); for the remaining cases immunohistochemistry (IHC) was done at the UNC Immunohistochemistry Core Laboratory. For DCIS cases, ER status was determined using IHC. PR status was not available for DCIS cases. For all DCIS and invasive breast cancers with available tissue sections, IHC was conducted for human epidermal growth factor receptor 2 (HER2) at UNC. Subtype definitions used for this study follow previously published studies.[15, 19, 23]

#### Variables of interest

Body mass index (BMI) and waist-to-hip ratio (WHR) were measured by a trained nurse at the time of interview (approximately 0–6 months after diagnosis), and the additional reproductive and lifestyle risk factors were self-reported through a nurse-administered, questionnaire (approximately 0–6 months after diagnosis). ‘Mode of detection’ for the cases was defined from questionnaires and categorized as self-detected, medically-detected, and screen-detected. Screen-detected women self-reported detection of their lesion through a routine screening mammogram. Screening mammography was reported as the mode of detection for 78% of Luminal A DCIS cases (n = 108) and 41% of Luminal A invasive breast cancers cases (n = 203).

#### Statistical analysis

Logistic regression was used to estimate odds ratios (ORs) and 95% confidence intervals (95% CIs) as the measure of association between risk factors and screen-detected Luminal A DCIS or invasive breast cancer, comparing each case group with the respective control group. The associations between the following risk factors and DCIS and invasive disease were estimated: age at menarche (≤13, >13), family history of breast cancer (yes, no), alcohol use (ever, never), smoking status (ever, never), any physical activity in the past three months (yes, no), BMI (continuous and <25, 25-<30, ≥30), WHR (<0.77, 0.77–0.83, ≥0.84), number of full term pregnancies (nulliparious,1–2, ≥3), age at first full term pregnancy (years) (nulliparous, <26, ≥26), breastfeeding (ever, never), and duration of OC use (years) (never, ≥3 months-≤5, >5-≤10, >10). All models were adjusted for the CBCS offset term to account for the sampling design of the study. We conducted tests of trend for BMI, WHR, number of full-term pregnancies, and duration of OC use using the ordinal variable and reporting the beta p-value. We estimated a p-interaction between DCIS and invasive breast cancer to determine whether there was evidence of statistical heterogeneity in the effect estimates between the two lesions for each risk factor.

Given that PR status was unavailable for DCIS cases, we performed sensitivity analyses removing Luminal A invasive cases that were ER- but PR+ (n = 76, 12%); ORs were not substantially altered when this subgroup was excluded (results not shown). Additional sensitivity analyses for risk factor associations including all Luminal A invasive breast cancers and DCIS lesions regardless of age or mode of detection are provided in S1 Table. All statistical analyses were done using SAS version 9.4 (SAS Institute, Cary NC). P-values were produced for a two-sided test with an alpha of 0.05 for statistical significance.

## Results

In the Carolina Breast Cancer Study, among women eligible for screening mammography (≥40 years of age), the prevalence of the Luminal A subtype was 62.3% among DCIS cases and 62.7% among invasive breast cancer cases. The majority of Luminal A DCIS were detected by mammography (77.7%); a smaller proportion of Luminal A invasive breast cancers were detected by mammography (41.1%). Compared to Luminal A invasive disease, a higher proportion of Luminal A DCIS cases were African American (DCIS: 25.9% vs Invasive: 16.8%) and were diagnosed among women with lower incomes (Table 1). The proportion of cases represented by postmenopausal women also differed between DCIS and invasive (DCIS: 75.9% vs Invasive: 83.7%, respectively).

**Table 1:** Socio-demographic characteristics of screen detected Luminal A DCIS and invasive breast cancer in the Carolina Breast Cancer Study, Phases 1–2 (1993–2001).

	DCIS ^θ^		Invasive Breast Cancer*	
	Controls N (%^)	Cases N (%^)	Controls N (%^)	Cases N (%^)
	424 (100.0)	108 (77.7)	1367	203 (41.1)
Age at diagnosis/ selection (yrs.)				
40–49	124 (41.8)	26 (24.1)	590 (44.6)	58 (16.4)
50–59	151 (33.4)	30 (27.8)	336 (29.8)	62 (35.3)
60–69	103 (18.1)	38 (35.2)	290 (17.2)	60 (35.7)
≥70	46 (6.7)	14 (13.0)	151 (8.5)	23 (12.6)
Menopausal status				
Pre	121 (40.7)	26 (24.1)	528 (41.1)	53 (16.3)
Post	303 (59.3)	82 (75.9)	839 (58.9)	150 (83.7)
Race				
White	356 (82.1)	83 (76.9)	751 (79.4)	124 (84.9)
African American	68 (17.9)	25 (23.1)	616 (20.6)	79 (15.1)
First degree family history of BC				
No	362 (87.1)	84 (80.0)	1150 (87.2)	152 (78.4)
Yes	56 (12.9)	21 (20.0)	167 (12.8)	46 (21.6)
Missing	6	3	50	5
Highest level of education				
Less than High School	61 (13.0)	8 (7.5)	280 (14.2)	36 (11.3)
High School Post High School	240 (56.6)	69 (63.9)	749 (56.5)	113 (59.9)
Collage or more	123 (30.5)	31 (28.7)	337 (29.2)	54 (28.8)
*Missing*	0	0	1	0
Income per year				
<$15,000	45 (9.7)	21 (20.8)	288 (15.4)	41 (13.1)
$15,000-$30,000	103 (25.3)	25 (24.8)	270 (19.9)	36 (17.8)
$30,000-$50,000	92 (24.5)	21 (20.8)	296 (24.9)	49 (32.7)
≥$50,000	146 (40.5)	34 (33.7)	388 (39.8)	64 (36.3)
Missing	38	7	125	13

^**θ**^ DCIS Luminal A lesions were defined as: HER2- and ER+ lesions.

*Invasive Luminal A lesions were defined as: HER2- (ER+ or PR+) stratified by mode of detection.

^All percentages weighted for sampling design

Case-control odds ratios (OR) and 95% confidence intervals (95% CI) for established breast cancer risk factors in association with screen-detected Luminal A DCIS and invasive breast cancer cases are presented in Table 2. Risk factor associations were not statistically different between DCIS and invasive disease; however, there were some similarities and differences in the patterns of association by tumor type. Similar patterns of association between tumor types were seen for family history of breast cancer [DCIS OR: 1.59, 95% CI (0.97, 2.89); Invasive OR: 2.03, 95% CI (1.39, 2.97)], increasing parity [≥3 vs 0 DCIS OR: 0.44, 95% CI (0.20, 0.97); Invasive OR: 0.55, 95% CI (0.33, 0.92)], and first full term pregnancy before age 26 [DCIS OR: 0.44, 95% CI (0.20, 0.98); Invasive OR: 0.55, 95% CI (0.33, 0.92)].

**Table 2:** Odds Ratios (OR) and 95% Confidence Intervals (95% CI) for screen detected Luminal A DCIS and invasive breast cancer cases (among women ≥40 years of age) compared to the respective controls from the Carolina Breast Cancer Study, Phases1-2 (1993–2001).

	Luminal A DCIS^θ^				Luminal A Invasive*			Invasive vs DCIS
	Controls N (%^)		Cases N (%^)	OR (95% CI)	Controls N (%^)	Cases N (%^)	OR (95% CI)	p-interaction^d^
**Number of full-term pregnancies**^a^								
Nulliparous		48 (13.6)	16 (14.8)	Ref	132 (10.0)	36 (16.9)	Ref	0.4
1–2		214 (51.6)	57 (52.8)	0.75 (0.37–1.53)	661 (52.8)	88 (44.0)	**0.56 (0.35–0.89)**	
≥3		162 (34.7)	35 (32.4)	**0.44 (0.20–0.97)**	574 (37.2)	79 (39.1)	**0.55 (0.33–0.92)**	
p-trend				0.02			0.07	
**Age at first full term pregnancy (years)**^a^								
Nulliparous		48 (13.6)	16 (14.8)	Ref	132 (10.0)	36 (16.9)	Ref	
<26		283 (64.4)	67 (62.0)	**0.44 (0.20–0.98)**	959 (66.5)	131 (63.7)	**0.55 (0.33–0.92)**	
≥26		93 (22.0)	25 (23.1)	0.44 (0.17–1.13)	273 (23.5)	36 (19.4)	0.59 (0.31–1.14)	
Missing		0	0		3	0		
**Family history of breast cancer**^a^								
No	362 (87.1)		84 (80.0)	Ref	1150 (87.2)	152 (78.4)	Ref	0.5
Yes	56 (12.9)		21 (20.0)	1.59 (0.87–2.89)	167 (167)	46 (21.6)	2.03 (0.39–2.97)	
Missing	6		3		1	5		
**Alcohol use**^a^								
Never	156 (34.6)		34 (31.5)	Ref	459 (30.1)	63 (28.7)	Ref	0.5
Ever	267 (65.4)		74 (68.5)	1.63 (0.96–2.79)	907 (69.9)	140 (71.3)	1.17 (0.81–1.68)	
Missing	1		0		0	0		
**BMI**^b^								
<25	142 (34.7)		45 (41.7)	Ref	426 (38.4)	61 (36.5)	Ref	0.08
25-<30	133 (30.2)		24 (22.2)	0.56 (0.31–1.00)	418 (30.1)	60 (33.9)	1.10 (0.74–1.65)	
≥30	149 (35.2)		39 (36.1)	0.76 (0.44–1.31)	523 (31.5)	82 (29.6)	1.30 (0.87–1.95)	
p-trend				0.30			0.19	
**WHR**^a^								
<0.77	129 (32.7)		29 (26.9)	Ref	416 (36.9)	40 (23.5)	Ref	0.1
0.77–0.83	148 (34.3)		41 (38.0)	0.98 (0.54–1.77)	427 (31.7)	73 (40.5)	1.95 (0.26–3.04)	
≥0.84	147 (33.1)		38 (35.2)	0.82 (0.42–1.60)	524 (31.4)	90 (36.0)	**1.99 (1.25–3.18)**	
p-trend				0.54			<0.01	
**Duration of oral contraceptive use (years)**^a^								
never	154 (29.5)		46 (43.9)	Ref	540 (32.6)	88 (45.9)	Ref	0.07
≥3 months-≤5	149 (38.3)		41 (38.3)	1.21 (0.67–2.18)	501 (40.1)	57 (27.2)	0.98 (0.64-.148)	
>5-≤10	71 (19.9)		12 (11.2)	0.66 (0.29–1.53)	195 (16.4)	34 (15.5)	1.57 (0.95–2.61)	
>10	48 (12.3)		8 (7.5)	0.70 (0.39–1.74)	120 (10.8)	24 (11.5)	**1.80 (1.02–3.17)**	
Missing	2		1		11	0		
p-trend				0.27			0.02	
**Smoking status**^a^								
Never	231 (55.7)		65 (60.2)	Ref	730 (51.2)	99 (41.8)	Ref	0.1
Ever	193 (44.3)		43 (39.8)	0.67 (0.41–1.09)	637 (48.8)	104 (58.2)	1.12 (0.80–1.56)	
**Age at menarche**^a^								
≤13	335 (80.0)		83 (76.6)	Ref	998 (75.1)	155 (75.1)	Ref	0.5
>13	89 (20.0)		25 (23.1)	1.12 (0.66–1.92)	362 (24.9)	48 (24.9)	0.89 (0.61–1.27)	
Missing	0		0		7	0		
**Ever lactated**^c^								
No	210 (56.1)		48 (52.2)	Ref	700 (57.3)	100 (58.3)	Ref	0.1
Yes	166 (43.9)		44 (47.3)	1.02 (0.62–1.67)	535 (42.7)	67 (41.7)	0.71 (0.50–1.02)	
**Physical activity**^a^								
No	182 (45.4)		46 (42.6)	Ref	651 (45.2)	104 (47.4)	Ref	0.5
Yes	241 (54.6)		62 (57.4)	1.01 (0.63–1.62)	714 (54.8)	99 (52.6)	0.79 (0.58–1.09)	

^θ^DCIS Luminal A lesions were defined as: HER2- and ER+ lesions.

*Invasive Luminal A lesions were defined as: HER2- (ER+ or PR+).

^All percentages weighted for study sampling design

^a^Full model included: offset term, age (continuous) and race (African American, non-African American), family history (yes, no), alcohol use (ever, never), smoking (ever, never), oral contraceptive use (ever, never), number of full term pregnancy (0,1–2, ≥3), breastfeeding (ever, never), age at menarche (≤13, >13 years), BMI (continuous), postmenopausal status (pre, post)

^b^Full model included: offset term, age, race, family history, alcohol use, smoking, oral contraceptive use, number of full term pregnancy, breastfeeding, age at menarche, postmenopausal status

^c^Full model (among parous women only) included: offset term, age, race, family history, alcohol use, smoking, oral contraceptive use, breastfeeding, age at menarche, postmenopausal status

^d^Interaction models adjusted for: offset term, age, and race

Differences in direction of association were present for a number of risk factors. Ever breastfeeding was not associated with DCIS [OR: 1.02, 95% CI (0.62–1.67)], but was weakly inversely associated with invasive breast cancer [OR: 0.71, 95% CI (0.50, 1.02)]. Higher WHR (≥0.84 versus <0.77) displayed an inverse association with DCIS [OR: 0.82, 95% CI (0.42, 1.60)], but was positively associated with invasive breast cancer [OR: 1.99, 95% CI (1.25, 3.18)]. Likewise, being obese (BMI≥30 vs <25) was inversely associated with DCIS [OR: 0.77, 95% CI (0.44, 1.31)] and positively associated with invasive breast cancer [OR: 1.30, 95% CI (0.87, 1.95)]. Oral contraceptive (OC) use for more than 10 years was differentially associated with DCIS and invasive breast cancer, with a weakly protective association for DCIS and strongly associated with invasive disease [DCIS OR: 0.70, 95% CI (0.39, 1.74); Invasive OR: 1.80, 95% CI (1.02, 3.17)]. Physical activity was inversely associated with invasive disease and was not associated with DCIS.

Smoking and alcohol drinking showed stronger associations with DCIS than invasive disease. Ever drinking alcohol was positively associated with DCIS [OR: 1.63 95% CI (0.96–2.79)], but the association was attenuated for invasive breast cancer [OR: 1.17 95% CI (0.81, 1.68)]. Ever smoking was inversely associated with DCIS but not invasive disease [DCIS OR: 0.67 95% CI (0.41, 1.09); Invasive OR: 1.12 95% CI (0.80, 1.56)].

Additional analyses, including all cases of Luminal A DCIS and Luminal A invasive breast cancer, regardless of age at diagnosis or mode of detection, are presented in S1 Table. To evaluate the role of screen-detection in these association, we stratified findings on mode of detection. Overall, risk factor associations for Luminal A DCIS were similar in both magnitude and direction to those observed among screen-detected lesions; effect estimates for Luminal A invasive breast cancers were generally similar in magnitude and direction, but slightly attenuated in medically-detected cases compared to those for screen-detected lesions.

## Discussion

Using data from the population-based Carolina Breast Cancer Study Phases 1–2, we found limited evidence that risk factor profiles may differ for screen-detected Luminal A DCIS compared to screen-detected Luminal A invasive breast cancer. Although no statistically significant interactions were detected, differences in the direction of association were observed for measures of adiposity (BMI and WHR) and oral contraceptive use for DCIS and invasive disease. Ever smoking was inversely associated with DCIS, but not invasive disease. We also observed that ever breastfeeding and physical activity were inversely associated with risk of invasive disease, but not DCIS. Risk factor profiles were similar between the two groups for family history of breast cancer, number of full-term pregnancies, and age at first full term pregnancy.

Many of the associations we observed track with published literature reports. As reported previously in the literature by Kabat *et al*. (2010) and Trentham-Dietz *et al*. (2007), we observed an inverse relationship between ever smoking and risk of Luminal A DCIS.[24, 25] This relationship may be causal in nature or may be a result of lower smoking levels among women who attended mammography screening. For invasive disease, we observed a slightly elevated, though non-significant, association similar in magnitude to that reported by Young *et al*. (2009) and Furberg *et al*. (2002)[26, 27] and by previous papers in the CBCS1/2[28] and the AMBER consortium.[29] Adiposity measures showed complex relationships with risk. The literature on BMI and risk of DCIS are consistent with our results in that high BMI has been associated with decreased DCIS risk[30–32] and increased risk of invasive disease, particularly among postmenopausal women.[33, 34] We saw a similar pattern for WHR. Previous literature suggests a positive association between WHR and invasive disease, but a null association for DCIS.[35] Differential patterns of association for OC use and breastfeeding were also supported by previous literature.[15, 23, 30, 36–43] An advantage of the current analysis is that the comparison between DCIS and invasive disease were conducted within a single population-based, well-defined and racially diverse study.

A previous CBCS analysis investigated differences between DCIS and invasive breast cancer wherein all DCIS were stratified by pathological subtype (comedo vs. non-comedo) and found comedo DCIS to have similar risk factor profiles to invasive breast cancer, whereas non-comedo DCIS may represent a distinct etiology.[23] In more recent years, a molecular subtyping of breast cancer has become more predominant for describing clinical heterogeneity. Most Luminal A breast cancers are non-comedo, and therefore, the suggestion of distinct etiology from the previous study parallels what we find here for molecular subtype. Screen-detected Luminal A invasive breast cancer is the ideal comparison group for Luminal A DCIS lesions because mammography is associated with other potential confounders (obesity, breastfeeding, etc.)[44–46], and the majority of DCIS diagnoses are screen-detected.

Our study has some limitations. First, we had limited sample size to investigate the interactions between DCIS and invasive disease and most of our interaction tests were not statistically significant. Second, we used IHC markers to act as a surrogate for Luminal A subtype. Although progesterone receptor (PR) was assessed for invasive and not DCIS cases, it was uncommon for invasive breast cancer in the CBCS to be PR positive and ER negative and therefore differentiation between subgroups of hormone receptor positive cancers was not possible. However, sensitivity analyses showed that exclusion of invasive breast cancers that were only PR positive did not substantially alter associations. Third, we acknowledge potential misclassification of the self-reported mode of detection, because this data was not confirmed by medical records or mammography history. Additionally, we do not expect any recall bias for lifestyle factors to differentially influence DCIS or invasive cases. We do not expect differential misclassification of mode of detection by DCIS or invasive breast cancer. By restricting our analysis to screen-detected invasive breast cancer and screening-eligible controls we have focused on a smaller subset of tumors, and our findings may not generalizable to other subtypes (i.e. Basal-like) and ages (e.g. breast cancer before age 40 years). However, our analysis has maximized internal validity to compare DCIS and invasive disease. Finally, analyses such as ours are helpful in identifying etiologic differences between diseases but are not well-suited to identify biologic mechanisms.

The widespread use of mammography has increased detection of both DCIS lesions and invasive breast cancers over the past decades.[1, 2] Identifying risk factors shared by DCIS and invasive breast cancer that may be associated with a higher likelihood of progression to invasive disease remains an important challenge for reducing the breast cancer burden. While it is difficult to determine the induction periods for breast cancer risk factor associations, performing studies on precursor lesions may help to elucidate risk factors associated with progression. The current analysis suggests that progression from DCIS to invasive disease may be associated with oral contraceptives and adiposity.

## Supporting information

S1 Table Displays odds ratios (OR) and 95% confidence intervals (95% CI) for risk factors for Luminal A DCIS and invasive breast cancer among all cases regardless of mode of detection or age from the Carolina Breast Cancer Study, Phases 1–2 (1993–2001). (DOCX)
