# Supplementary material for: Risk factors for Luminal A ductal carcinoma in situ (DCIS) and invasive breast cancer in the Carolina Breast Cancer Study
Source: PLoS One. 2019 Jan 25;14(1):e0211488. doi: 10.1371/journal.pone.0211488 (PMC6347264; doi:10.1371/journal.pone.0211488)
Supplement: S1 Table — (DOCX) [file pone.0211488.s001.docx]

**Supplemental Table 1. Odds Ratios (ORs) and 95% CIs for risk factors for Luminal A DCIS and invasive breast cancer, regardless of mode of detection or age, from the Carolina Breast Cancer Study, Phases 1-2 (1993-2001)**

|  | **Luminal A DCIS^θ^** | | | **Luminal A* Invasive** | | | **Invasive vs DCIS** |
| --- | --- | --- | --- | --- | --- | --- | --- |
|  | **Controls** | **Cases** | **OR (95% CI)** | Controls | **Cases** | **OR (95% CI)** | **p-interaction^d^** |
| **Mean age at study enrollment (SD)** | 54.5 (10.3) | 55.7 (11.1) |  | 52.0 (11.5) | 52.4 (11.2) |  |  |
| **Age at menarche**^a^ | |  |  |  |  |  |  |
| ≤13 | 363 (82.3) | 113 (74.8) | Ref | 1141 (69.5) | 470 (74.4) | Ref | 0.53 |
| >13 | 95 (17.7) | 38 (25.2) | 1.20 (0.75-1.90) | 415 (30.5) | 155 (25.6) | 0.86 (0.69-1.08) |  |
| Missing | 0 | 1 |  | 8 | 0 |  |  |
| **Family history of breast cancer** ^a^ | | |  |  |  |  |  |
| No | 394 (89.4) | 118 (80.3) | Ref | 1328 (82.2) | 505 (83.3) | Ref | 0.70 |
| Yes | 58 (10.6) | 29 (19.7) | 1.67 (0.99-2.84) | 183 (17.8) | 101 (16.7) | 1.49 (1.13-1.95) |  |
| Missing | 6 | 5 |  | 53 | 19 |  |  |
| **Alcohol use** ^a^ | | |  |  |  |  |  |
| Never | 160 (28.2) | 45 (29.6) | Ref | 506 (26.0) | 187 (30.5) | Ref | 0.28 |
| Ever | 297 (71.8) | 107 (70.4) | 1.52 (0.94-2.46) | 1057 (74.0) | 438 (69.5) | 1.06 (0.84-1.34) |  |
| Missing | 1 | 0 |  | 1 | 0 |  |  |
| **Smoking status** ^a^ | | |  |  |  |  |  |
| Never | 247 (57.3) | 82 (53.9) | Ref | 840 (47.8) | 316 (47.7) | Ref | 0.65 |
| Ever | 211 (42.7) | 70 (40.1) | 0.87 (0.57-1.33) | 724 (52.2) | 309 (52.3) | 1.11 (0.91-1.37) |  |
| Missing | 0 | 0 |  | 0 | 0 |  |  |
| **Physical activity** ^a^ | | |  |  |  |  |  |
| No | 196 (44.3) | 68 (45.0) | Ref | 750 (51.0) | 298 (44.9) | Ref | 0.65 |
| Yes | 261 (55.7) | 83 (55.0) | 0.96 (0.63-1.45) | 814 (49.0) | 327 (55.1) | 0.95 (0.78-1.16) |  |
| Missing | 1 | 1 |  | 0 | 0 |  |  |
| **BMI** ^b^ |  |  |  |  |  |  |  |
| <25 | 160 (41.7) | 65 (42.8) | Ref | 502 (43.3) | 235 (39.5) | Ref | 0.23 |
| 25-<30 | 137 (26.5) | 32 (21.1) | 0.56 (0.34-0.95) | 472 (26.4) | 171 (31.9) | 0.83 (0.64-1.06) |  |
| ≥30 | 161 (31.7) | 55 (36.2) | 0.78 (0.48-1.25) | 590 (30.3) | 219 (28.6) | 0.92 (0.72-1.18) |  |
| Missing |  | 0 |  | 0 | 0 |  |  |
| p-trend |  |  | 0.27 |  |  | 0.51 |  |
| **WHR** ^a^ |  |  |  |  |  |  |  |
| <0.77 | 142 (36.3) | 47 (30.9) | Ref | 502 (44.2) | 170 (26.8) | Ref | 0.09 |
| 0.77-0.83 | 162 (33.6) | 50 (32.9) | 0.78 (0.46-1.29) | 484 (27.7) | 211 (37.2) | 1.55 (1.19-2.02) |  |
| ≥0.84 | 154 (30.1) | 55 (36.2) | 0.81 (0.45-1.45) | 578 (28.1) | 244 (26.0) | 1.68 (1.26-2.23) |  |
| Missing | 0 | 0 |  | 0 | 0 |  |  |
| p-trend |  |  | 0.50 |  |  | <0.01 |  |
| **Number of full term pregnancies** ^a^ | | |  |  |  |  |  |
| Nulliparous | 56 (17.9) | 25 (16.4) | Ref | 174 (22.6) | 104 (15.1) | Ref | 0.60 |
| 1-2 | 237 (52.5) | 77 (50.7) | 0.76 (0.41-1.42) | 776 (49.6) | 294 (47.6) | 0.74 (0.54-1.01) |  |
| ≥3 | 164 (29.6) | 50 (32.9) | 0.51 (0.25-1.01) | 614 (27.8) | 227 (37.3) | 0.77 (0.55-1.08) |  |
| Missing | 0 | 0 |  | 0 | 0 |  |  |
| p-trend |  |  | 0.03 |  |  | 0.30 |  |
| **Age at first full term pregnancy (years)** ^a^ | | |  |  |  |  |  |
| Nulliparous | 56 (17.9) | 25 (16.4) | Ref | 174 (22.6) | 104 (15.1) | Ref | 0.69 |
| <26 | 297 (58.7) | 88 (57.9) | 0.49 (0.25-0.99) | 1057 (54.7) | 380 (61.3) | 0.77 (0.55-1.08) |  |
| ≥26 | 105 (23.4) | 39 (25.7) | 0.62 (0.28-1.43) | 330 (22.7) | 138 (23.6) | 0.88 (0.58-1.34) |  |
| Missing | 0 | 0 |  | 3 | 3 |  |  |
| p-trend |  |  | 0.32 |  |  | 0.30 |  |
| **Ever lactated** ^c^ | | |  |  |  |  |  |
| No | 217 (50.9) | 65 (51.2) | Ref | 776 (53.6) | 293 (54.8) | Ref | 0.29 |
| Yes | 185 (49.1) | 62 (48.8) | 1.06 (0.69-1.65) | 614 (46.4) | 228 (45.2) | 0.83 (0.66-1.03) |  |
| Missing | 0 | 0 |  | 0 | 0 |  |  |
| Missing |  | 1 |  | 11 | 2 |  |  |
| **Duration of oral contraceptive use (years)** ^a^ | | |  |  |  |  |  |
| never | 156 (23.3) | 59 (39.1) | Ref | 572 (23.5) | 219 (40.9) | Ref | 0.11 |
| ≥3months-≤5 | 161 (35.8) | 62 (41.1) | 1.23 (0.73-2.05) | 570 (43.6) | 223 (32.7) | 1.34 (1.03-1.75) |  |
| >5-≤10 | 82 (25.7) | 18 (11.9) | 0.60 (0.29-1.24) | 242 (20.3) | 102 (15.3) | 1.31 (0.94-1.83) |  |
| >10 | 57 (15.2) | 12 (7.9) | 0.65 (0.30-1.40) | 169 (12.5) | 79 (11.1) | 1.46 (1.02-2.10) |  |
| Missing | 2 | 1 |  | 11 | 2 |  |  |
| p-trend |  |  | 0.11 |  |  | 0.04 |  |

^θ^DCIS Luminal A lesions were defined as: HER2- and ER+ lesions.

*Invasive Luminal A lesions were defined as: HER2- and ER+ or PR+.

^All percentages weighted for study sampling design

^a^Full model included: offset term, age (continuous) and race (African American, non-African American), family history (yes, no), alcohol use (ever, never), smoking (ever, never), oral contraceptive use (ever, never), number of full term pregnancy (0,1-2, ≥3), breastfeeding (ever, never), age at menarche (≤13, >13 years), BMI (continuous), postmenopausal status (pre, post)

^b^Full model included: offset term, age, race, family history, alcohol use, smoking, oral contraceptive use, number of full term pregnancy, breastfeeding, age at menarche, postmenopausal status

^c^Full model (among parous women only) included: offset term, age, race, family history, alcohol use, smoking, oral contraceptive use, breastfeeding, age at menarche, postmenopausal status

^d^Reduced model adjusted for: offset term, age (continuous) and race (African American, non-African American)
